# The Role of Clusterin Transporter in the Pathogenesis of Alzheimer’s Disease at the Blood–Brain Barrier Interface: A Systematic Review

**DOI:** 10.3390/biom12101452

**Published:** 2022-10-10

**Authors:** Muhammad Mazhar Fareed, Maryam Qasmi, Shaan Aziz, Elisabeth Völker, Carola Yvette Förster, Sergey Shityakov

**Affiliations:** 1 Department of Computer Science, School of Science and Engineering, Università Degli Studi di Verona, Via dell’Artigliere, 8, 37129 Verona, Italy or; 2 Department of Bioinformatics and Biotechnology, Faculty of Life Sciences, Government College University, Faisalabad 38000, Pakistan or or; 3 Department of Anaesthesiology, Intensive Care, Emergency and Pain Medicine, Würzburg University Hospital, 97070 Würzburg , Germany; 4 Laboratory of Chemoinformatics, Infochemistry Scientific Center, ITMO University, 195251 Saint-Petersburg, Russia

**Keywords:** clusterin transporter, *apolipoprotein J*, Wnt signaling, Alzheimer’s disease, AD pathogenesis, blood–brain barrier

## Abstract

Alzheimer’s disease (AD) is considered a chronic and debilitating neurological illness that is increasingly impacting older-age populations. Some proteins, including clusterin (*CLU* or *apolipoprotein J*) transporter, can be linked to AD, causing oxidative stress. Therefore, its activity can affect various functions involving complement system inactivation, lipid transport, chaperone activity, neuronal transmission, and cellular survival pathways. This transporter is known to bind to the amyloid beta (Aβ) peptide, which is the major pathogenic factor of AD. On the other hand, this transporter is also active at the blood–brain barrier (BBB), a barrier that prevents harmful substances from entering and exiting the brain. Therefore, in this review, we discuss and emphasize the role of the *CLU* transporter and *CLU*-linked molecular mechanisms at the BBB interface in the pathogenesis of AD.

## 1. Introduction 

Due to the high global prevalence (46.8 million people) of Alzheimer’s disease (AD), AD as a degenerative neurological condition attracts a lot of attention from the scientific community. About 60–80 percent of those with dementia have AD, which is very common for this pathological condition [1]. Every 6.3 years, the prevalence of all dementias increases from 3.9 per 1000 people aged 60–90 years to 104.8 per 1000 people aged beyond 90 years, making aging the biggest risk factor for AD. People over the age of 80 are expected to have a 40% incidence rate, while those over 65 are expected to have only a 10% incidence rate [2]. Pre-clinical detection and treatment for AD are urgently needed due to its high financial burden and patient suffering. AD is characterized by the formation of extracellular amyloid beta (Aβ), neuroinflammation, aggregation of intracellular tau protein, cerebral atrophy, and loss of neuronal connections. Indeed, uncommon variants of familial autosomal dominant AD (FAD) begin with initial conditions at an age range of thirty to sixty and are caused by mutations in the presenilin *PSEN-1, PSEN-2*, and *amyloid precursor protein (APP)* genes [3,4]. There are several genes associated with an increased risk of AD, including APOE-4, which has been also linked to FAD.

Clusterin (*CLU*), also known as *apolipoprotein J (APOJ)*, is a disulfide-linked heterodimeric multifunctional chaperone molecule that is 75 to 80 kDa [5]. The *CLU* gene on chromosome 8 encodes the *CLU* protein, which has been linked to an increased risk of developing late-onset AD (LOAD) in humans [6]. *CLU* is a secreted mammalian chaperone, which might influence severe pathology associated with extracellular protein aggregation and folding [7]. Some resent insights gave evidence that, in contrast to other chaperones, *CLU* may play a vital role in maintaining proteostasis, both within and outside cells and tissues, in biological fluids. Being a multifunctional glycoprotein, *CLU* has been demonstrated to also be involved in multiple physiological and pathophysiological processes, including AD [8].

On the other hand, cell proliferation, migration, and differentiation are all regulated by the Wnt–catenin signaling system, and Wnt proteins are essential for the survival of mammalian cells. A minor fraction (9%) of AD patients has a relation to the *CLU* gene [9]. Moreover, elevated *CLU* levels in the cerebrospinal fluid (CSF) and the brain of AD patients were also detected. *CLU* has also been linked to several clinical and pathological conditions, including rapid disease progression, severity, and cerebral atrophy in AD convalescents [10]. Additionally, other proteins known as *APOE* (apolipoprotein E) have been shown to play an important role in a development of AD, together with *CLU* [11]. However, the role of *CLU* in the AD pathogenesis has yet to be fully defined. Therefore, in this review we discuss possible involvement of *CLU* and its signaling pathways that might be triggered due to AD mainly in the brain and at the blood–brain barrier interface.

## 2. Attempts to Use CLU as an AD Biomarker

A study performed in 1990 found that increased amounts of messenger ribonucleic acid (mRNA) of *CLU* were located throughout areas of the cerebral matter (brain) of individuals affected by Alzheimer’s disease (AD) when compared to controls. Elevated *CLU* protein levels were found in the hippocampus and frontal cortex of post-mortem AD brains. Detection of *CLU* staining in senile plaques, Aβ deposits, neuropil threads, and neurons free of neurofibrillary tangles (NFTs)have been identified. However, in this study, *CLU* immunoreactivity was only observed in rare cases in neurons that contained NFTs [12]. Positron emission tomography imaging revealed that Alzheimer’s disease (AD) patients with elevated plasma *CLU* levels exhibited an increased fibrillar Aβ load in the entorhinal cortex (EC). Several alleles have also been linked to increased levels of *CLU* and decreased levels of *APOE* [9,13]. However, in a related study, researchers found the exact opposite effect: presence of the *APOE-4* allele significantly lowered the *CLU* level in the frontal lobe region of people with Alzheimer’s disease. *CLU* is detected in lipoprotein particles in the cerebrospinal fluid. Aside from the fact that the degree of *CLU* glycosylation can fluctuate and the *CLU* complexes with both Aβ and Aβ-fibrils, it is difficult to measure the level of *CLU* in cerebrospinal fluid [14]. There was no difference between AD patients and controls in previous studies, but subsequent investigations using current methodologies indicated that the *CLU* level in CSF of AD patients dramatically increased. Using both neuroimaging and proteomic methods, researchers discovered that plasma *CLU* levels are associated with criterion levels of the gravity of a disease/condition, cerebral atrophy (in the entorhinal cortex and inner (medial) region of the temporal lobe), and expeditious development of Alzheimer’s disease, indicating that *CLU* levels in plasma could serve as an AD biomarker. This was subsequently showcased by way of genetically modified mice with higher cerebral-related deficits, as well as the accumulation of Aβ within the brain [15,16]. Some studies have not found a difference between mRNA levels between AD patients and controls [17]. However, more recent investigations using current methodologies have observed a higher *CLU* level in the CSF (cerebrospinal fluid) of AD patients [18]. Using both neuroimaging and proteomic methods, researchers discovered that plasma *CLU* levels are associated with criterion levels of the gravity of a disease/condition, cerebral atrophy (in the entorhinal cortex and inner [medial] region of the temporal lobe), and expeditious development of Alzheimer’s disease, indicating that *CLU* levels in plasma could serve as an AD biomarker.

### 2.1. Metabolic and Toxic Consequences of CLU-Alpha–Beta Proteins

*CLU* has been linked to Aβ for the last two decades. Using a cellular model system, *CLU* was able to prevent the accumulation of Aβ in an in vitro investigation. LR-P2, the endocytic receptor of *CLU*, was identified and found to stimulate *CLU*-Aβ transport over the blood–CSF barrier and the blood–brain barrier (BBB) [19]. A wide range of downstream biological activity can be seen owing to the use of various Aβ species, incubation techniques, and aggregate protocols. *CLU*–Aβ interactions have been found to play a substantial role in the production and toxicity of amyloids in both in vitro and in vivo studies. Additionally, in vitro studies show that *CLU* can interact with Aβ-soluble forms (Aβ 1–40 and Aβ 1–42). Furthermore, the complex formation significantly reduces Aβ polymerization and aggregate formation. Soluble Aβ is protected from degradation by binding to *CLU* [16]. Using sandwich ELISA, researchers determined the concentrations of insoluble (guanidine-HCl-extracted) *CLU*, and Aβ, in areas of the brain with a predilection for amyloid anatomy (PH), the mid-frontal gyrus (MF), the medial area of the cerebral cortex (CC), as well as other parts with small or no anatomy (WM and thalamus), substantia alba (WM), and CC (TH). Areas with plaque pathology (the parietal cortex [PC], PH, -C, and MF]) had the highest levels of *CLU*, which roughly matched the regional Aβ distribution. This behavior was significantly more prevalent in AD patients than in the general population and was positively associated with Aβ [20].

**Figure 1:**
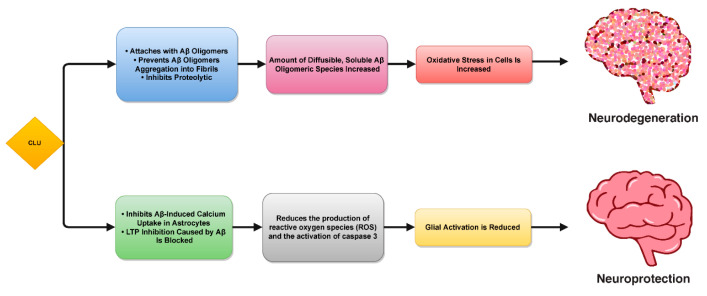
The molar ratio of *CLU* and Aβ was postulated to play a role in the dual effects of *CLU* on Aβ toxicity. Determining whether it is preferable to lower or raise *CLU* levels in humans is critical [13]. Changing the level of human *CLU* affects Aβ pathology, thus it is important to figure out whether and to what extent this may be done therapeutically to modify Aβ levels and toxicity, and whether or not this can be used to treat Alzheimer’s disease (AD) [15,20].

In AD patients, this behavior was significantly more prevalent than in the general population, and it was associated positively to Aβ42 and insoluble Aβ40. The largest concentration of *APOE-4* homozygotes and soluble *CLU* were found in the PC and MF regions, both of which had a higher severity of cerebral amyloid angiopathy. *CLU* was found to be unaffected in reduced amyloid anatomy parts, such as white matter and TH, and has been not linked with Aβ levels [20,21,22]. *CLU* levels and Aβ42 concentrations were found to have a strong positive correlation. A lower *CLU*/Aβ42 molar ratio was found in regions where Aβ plaque disease is more common, whereas this ratio was highest in areas with the lowest levels of insoluble Aβ42 [23]. *CLU* reduces Aβ aggregation and facilitates Aβ elimination under physiological circumstances [19,24]. The *CLU*/Aβ42 ratio in these regions witnesses a reduction, possibly having a significant effect on the deposition of Aβ in the tissue [22]. *CLU* might be associated with amyloid formation, where the soluble Aβ oligomers are mediated by *CLU* in vitro models [22]. It was also found that tiny diffusible Aβ oligomers induced by *CLU* corresponded to greater levels of neuronal damage in the CNS cells [25]. The aggregation process of Aβ can be disrupted as a result of implementation of anti-amyloid compounds [26].The aggregation process of Aβ can be disrupted, but this can have an impact on the damaging activities of amyloid compounds [27,28]. According to the results of one study, cells treated with AD CSF had a cytoprotective effect due to the combination of extracellular chaperones, including *CLU* [19]. The co-culture investigations showed that *CLU* maturation stopped Aβ-mediated astrocytic calcium absorption, which subsequently decreased reactive oxygen species formation and initiation involving caspase-3 [27]. Moreover, *CLU* also averts Aβ-mediated LTP (long-term potentiation) inhibition in hippocampal slices [29]. Injection of *CLU* with Aβ aggregates improved the Morris water maze performance in rat models and decreased neuronal deterioration along with glia activation [30]. Organotypic mouse brain slices, on the other hand, exhibited an increase in more soluble, diffusible oligomeric Aβ species and decreased fibril formation, both of which led to an increase in cellular oxidative damage and neurotoxicity (Figure 1) [19,31,32].

### 2.2. Aβ Role in Cerebral Amyloid Angiopathy Involving CLU and Wnt at the BBB 

Cerebral amyloid angiopathy (CAA) is a disease of the small vessels characterized by Aβ peptide deposits, cerebral microhemorrhages, and leakage of the blood–brain barrier. Due to the commonality of Aβ deposits, CAA is often associated with AD [33]. Two subtypes can be distinguished here: Type 1 is characterized by capillary Aβ deposits, and Type 2 by deposits in the leptomeningeal and cortical arteries. Impaired vascular perfusion and subsequently dementia are due to CAA type 1, which is also associated with the APOE-ɛ4 genotype. In addition, it was found that Aβ aggregation and amyloid fibril formation can be inhibited by *CLU*, and furthermore, Aβ clearance through the BBB is mediated by *CLU* via lipoprotein-related protein-2 (LRP2) [34]. Furthermore, *CLU* plays a role in the Aβ-DKK1 pathway concerning the Wnt signaling system. On the one hand, treatment with Aβ oligomers induces *CLU* expression and reduces protein secretion, which in turn leads to the induction of DKK1, removing the ligand LRP6 from the functional canonical receptor heterodimer. Reduced canonical Wnt signaling decreases P-gp expression in brain endothelial cells in AD patients, resulting in the absence of P-gp-mediated secretion of Aβ on the luminal side of the plasma membrane into the general circulation, which in turn increases retention in the brain. 

The network that links APP processing to Wnt signaling appears to become a pathological feedback loop in the case of AD. Suppressed Wnt signaling leads to increased amyloidogenic processing, further inhibiting Wnt signaling. 

The Wnt signaling pathway is central to the maintenance of the blood–brain barrier. As a result, insufficient Aβ clearance can lead to mechanical effects, such as reduced capillary blood flow, but can also have direct toxic effects on endothelial cells [35]. The proinflammatory response triggered by Aβ also plays a crucial role in BBB dysfunction associated with CAA. Here, on the one hand, there is an impairment of oxidative metabolism, coagulation status, and barrier properties, and on the other hand, there is an induction of the adaptive and innate immune system with activation of microglia and macrophages, as well as the support of B and T cell transmigration. The ensuing vicious cycle of decreased Aβ clearance, leading to acceleration of endothelial injury and increased perivascular inflammation, demonstrates the decisive role of the BBB in the pathogenesis of CAA [33].

### 2.3. Aβ Clearance Mechanism (CLU)

**Figure 2:**
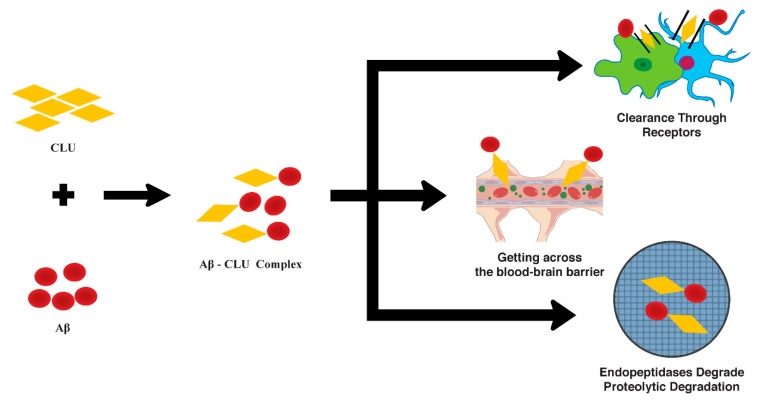
Alzheimer’s disease and the effects of *CLU* on Aβ clearance in AD.

Intracellular uptake and BBB delivery are two pathways that help to remove Aβ from the brain, contributing to *CLU* activity in AD. The brain’s parenchymal cells, such as neurons, astrocytes, and the microglial cells, together with the BBB and endopeptidase-provoked proteolytic breakdown, are all important mechanisms for Aβ removal [36]. The brain’s interstitial fluid is filtered by microglia and astrocytes assisting to remove Aβ. However, the role of *CLU* in these processes is yet to be elucidated. Some studies suggest that *CLU* plays a role in the clearance of Aβ by increasing internal *CLU* levels, decreasing secreted *CLU* levels, and increasing the development of Aβ fibrils in human astrocytes after Aβ treatment [36]. Furthermore, the incubation of pre-aggregated Aβ and *CLU* in human primary astrocyte cultures and fibril-rich preparations in microglia inhibited amyloid uptake from oligomer-enriched preparations [36]. Using macrophage-like U-937 cells, a study found that co-incubation with *CLU* and CSF from AD patients improved Aβ clearance from the supernatant [37]. In addition, the Aβ internalization has greater efficiency when it is formulated with *CLU* or low-density lipoproteins (LDL) [37] When Aβ is complexed with *CLU* in vivo, the lipoprotein receptor-related protein-2 could also be found to facilitate Aβ clearance [37]. Additionally, *CLU* is implicated in the monitoring of Aβ entrance over the BBB in the BBB model where endothelial cells are grown on the permeable substrate separating apical and basolateral partitions [8,38]. Furthermore, Aβ internalization has greater efficiency during the time a complex is formulated with *CLU* or low-density lipoproteins (LDL). TREM-2 is also a risk factor for Alzheimer’s disease, much as *CLU*. These data, taken together, imply that multiple-risk genes are linked to AD pathogenesis [39]. A complex of *CLU* and Aβ could promote the transport of Aβ from the basolateral to the apical compartment, but the *CLU* trafficking between these compartments was hampered by the blockage of lipoprotein receptor-related protein 1 and 2 [40]. The intracellular levels of the APP protein and the transport of radio-labelled Aβ were both increased when the exogenous *CLU* was added to the culture [41]. By modulating Aβ transport, *CLU* might play a role in the local clearance of soluble Aβ from the ISF (Figure 2) [40]. This could be achieved via endocytosis of the *CLU*–Aβ complex to nerve cells. Moreover, *CLU* and Aβ co-incubation could increase the Aβ uptake and degradation of *CLU*–Aβ complexes in the teratocarcinoma F9 cells [42,43].

## 3. The Role of *CLU* in AD-Signaling Channels (Neuro-Inflammatory and Wnt Signaling)

There is a relationship between *CLU* (clusterin (apolipoprotein *J*)) and Aβ in vivo through the utilization of a model organism of amyloidosis with a *CLU* deficiency. Yet, the before-mentioned research was insufficient in elucidating *CLU*’s anti-amyloidogenic activity. The establishment of the plaque had been reduced in *CLU*-knockout (KO) PD-APP models integrating mice [44,45], when compared to *CLU*-expressing PD-APP animal models. The lack of clusterin reduced the surrounding neuritic-dystrophy plaques formed in almost the same mouse model, suggesting that *CLU* has an amyloidogenic action. As contrasted with the complete elimination of *APOE* separately in PD-APP mice models, it was visible that a full suppression of both *APOE* and clusterin raised amyloid deposition. It initially began with AD pathogenesis and Aβ production These results suggested that *CLU* and *APOE* may collaborate to reduce amyloid deposition in these mice models, highlighting the complexity of understanding apolipoprotein J and Aβ interactions in vivo. *CLU* knockout was observed to change alpha–beta deposition from plaques to accumulation in the cerebrovasculature [46] in APP/PS-1 mouse models, which increased cerebral amyloid angiopathy, while reducing inflammation and bleeding [47]. In addition, a study found that the build-up of oligomeric properties of Aβ in synapses maintaining clusterin is elevated within *APOE-4* holders. Similar to *APOE-4*, apolipoprotein J, apolipoprotein E are significant predictors of Alzheimer’s disease and may play a role in the pathogenesis of atherosclerosis [36,47,48,49]. Other research examined whether clusterin genetic variation was linked to AD or atherosclerosis-related disorders. In addition, the analysis revealed that the T allele of *CLU* rs-9331896 was associated with an increased risk of AD in general, but not with an elevated risk of atherosclerosis-associated disorders such as ischemic cerebrovascular disease, ischemic heart disease, or vascular dementia [5]. There was no link found between the ε4 allele and *CLU* rs-9331896 [50] in forecasting Alzheimer’s disease, irrespective of what other kind of outcomes were investigated. With the exception of prior research, this one uses a mix of individual risk factor elucidations, the exact causal ε4 allele [50], as well as various subcategories corresponding to dementia and atherosclerosis-related [51] termination points, as well as multiple subtypes of dementia and atherosclerosis-related endpoints. This is in addition to multiple subtypes of dementia and atherosclerosis-related endpoints. Inflammation of the brain is a key feature of Alzheimer’s disease pathogenesis. Chronic therapy containing non-steroidal anti-inflammatory medicines decreases Alzheimer’s disease risk and may also delay development, implying that inflammatory processes are closely connected with AD-related neurodegenerative qualities [52]. Local activation of the complement system, astrocytes, and microglia-based structure is discovered to be induced by an intrinsic immunological response and subsequent neuroinflammation [53]. Furthermore, the resulting local pro-inflammatory pathways might promote the production of cytokines, potentially cytotoxic chemicals, and other associated compounds, which can eventually lead to neurodegeneration [54]. Clusterin has been connected to immunity and neuroinflammation in a variety of mechanisms, including negative Nuclear Factor-kappa B regulation and complement activation, according to multiple studies. Modulation, microglial inflammation, and bidirectional inhiation, along with primary pro-inflammatory cytokines possessing interleukin 6 (IL-6), resulting in a transformation growth factor (TGF-β1), as well as a TNF-α, is defined as tumor necrosis. For a greater understanding of the impact of *CLU* on the inflammatory processes implicated in AD development, more research is needed, with the inclusion of precise pharmacological and genetic alterations of specific agitational systems [55]. Apolipoprotein J and Aβ interacted directly, resulting in downstream processes [56]. Moreover, *CLU* has been linked to Wnt signaling; the role of this signal transduction pathway in the development of Alzheimer’s disease has been extensively researched. After neurons were treated with Aβ, they induced a neurotoxic reflex and an increase in the amount of Dickkopf-1 (DKK-1) (a Wnt signaling antagonist) [55,57,58]. This in turn led to synaptic loss, greater tau phosphorylation, and activation of glycogen synthase kinase 3β (GSK-3β) [56]. *CLU-*KO suppressed the Aβ-induced elevation in DKK-1 expression in mouse primary cortical neurons and also protected the cells against the Aβ-mediated neurotoxicity. Tracking the Aβ simulation of the cells, the intracellular amount of clusterin level increased, while the extracellular quantity of released clusterin level decreased [59]. This is consistent with the findings in astrocytes. Surprisingly, all discovered changes took place in less than thirty minutes, with the reduction integrating with *CLU* mRNA expression. The aforementioned would dictate that *CLU* changes are post-transcriptional and primarily occur as a result of changes in *CLU* secretion [60]. Additional research conducted within this field resulted in the hypothesis of a shift in Wnt signaling at the unorthodox Wnt-PCP-JNK tract. It was followed by the mobilization of downstream transcription features increased by a combination of Aβ along with DKK-1, which might be prevented by knocking down apolipoprotein J [60]. Conversely, when these genes were muted individually, they shielded the cultures from Aβ-mediated neuronal cell death (KLF-10 and EGR-1) and aided as a component in the rebuilding of phosphorylated tau (p-tau) to questionable proportions (KLF-10 and EG-R1) (NAB-2 and EGR-1) [46]. The length of neurite surviving Aβ damage has been shown to maintain a retainment within the *CLU*-KO cells in a project creating human neurons produced from induced pluripotent stem cells. This would only reinforce the evidence for *CLU*-mediated Aβ toxicity [44,61]. Resulting in inaccurate Aβ plaques and clusterin encompassed through p-tau-positive dystrophic neurites accompanied by p-tau deposits. These show themselves to be formed in the temporal cortex of people with Alzheimer’s disease, which has also been verified in studies. Secondly, *CLU* has been discovered to have been co-localized as complementary to NFTs inside the AD-EC. Moreover, neurons with intracellular neurofibrillary tangles had higher apolipoprotein J expression [44]. Tau and p-tau levels were shown to be higher in rats receiving intracerebral *CLU* injections, as well as primary cortical neurons in mice [50]. These observations contradict earlier findings, which showed an increased number of NFT-free suggests *CLU* in the frontal, temporal, and entorhinal cortices of AD patients, and that apolipoprotein J was rarely found in neurons with NFTs [50,52,62].

On the other hand, CLU has been well-characterized to play a pivotal role in autophagy, which is a major catabolic pathway in which the cell degrades macromolecules and damaged organelles [7]. According to recent insights, *CLU* probably exits the secretory pathway by the unknown mechanism to thereafter re-enter the cell after the secretion process [8]. *CLU* binds and interacts with Aβ, thereby modifying aggregation patterns. This process also promotes Aβ clearance so that a neuroprotective role for *CLU* is inferred [63,64]. Additionally, clusterin attenuates Aβ toxicity, as the knockdown of *CLU* in rodent and human iPSC-derived neurons is neurotoxic [8]. With a prominent extracellular chaperone function, additional roles have been discussed for clusterin, including lipid transport and immune modulation. It is involved in pathways common to several diseases such as cell death and survival, oxidative stress, and proteotoxic stress. Although clusterin is normally a secreted protein, it has also been found intracellularly under certain stress conditions [7].

## 4. The Role of Wnt/β-Catenin Signaling and Neurogenesis in AD

**Figure 3:**
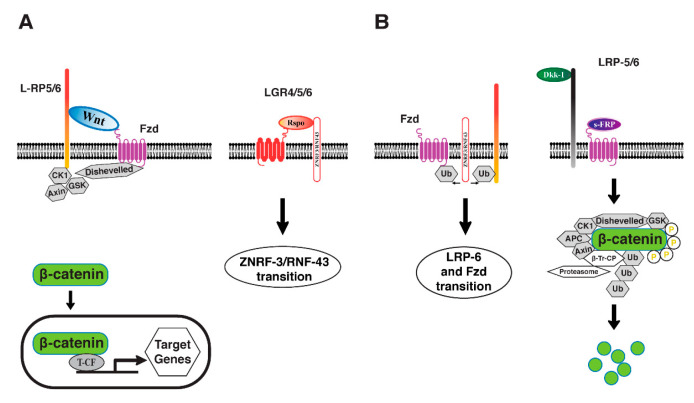
The route of Wnt/-catenin signaling. (**A**) Phosphorylation and degradation of beta-catenin are prevented when Wnt proteins bind to LR-P-5/6 and F-ZD, resulting in catenin stability, accumulation, and nuclear translocation, as well as pathway activation. (**B**) Once the Wnt antagonists Dkk-1, SOST, and s-FRP prevent Wnt binding to receptors, beta-catenin is phosphorylated by Ck-1 and GS-K-3 and then destroyed by the 26S proteasome. Rspo proteins and their receptors, LG-R-4, LG-R-5, and LG-R-6, positively control Wnt receptor Fz-d and Wnt co-receptor LR-P-5/6 at the cell surface, while E-3 ubiquitin ligases RN-F-43 and ZNR-F-3 negatively regulate them.

Wnt proteins are released glycoproteins that promote the canonical Wnt/ β -catenin signal transduction by binding to the exterior cysteine-rich domain of the Frizzled (Fzd) receptor family and Wnt co-receptor low-density lipoprotein receptor-related protein 5 (LRP-5) or LRP-6 [46,51]. Glycogen synthase kinase 3β (GSK3β) is inhibited and cytosolic β-catenin is stabilized when Wnt binds to the Fzd/LRP5/6 response element. The stabilized β-catenin eventually translocate to the nucleus, where it interacts with the T cell factor/lymphoid-enhancing factor (TCF/ LEF). This eventually triggers the production of certain target genes [51]. Various secreted proteins and receptors govern Wnt/β-catenin signaling in the cell membrane. Although the extracellular molecule, R-spondin (Rspo), and its nerve cells’ leucine-rich resumption comprising G protein-coupled receptor 4/5/6 (LGR4/5/6) promote LRP5/6 degradation, R-spondin (Rspo) and its receptors’ leucine-rich repeat containing G protein-coupled receptor 4/5/6 (LGR4/5/6) stimulate ZNRF-3/RNF-43 revenue, attempting to make LRP5/6 available on the cell surface for activation of the Wnt/β-catenin signaling pathway [61]. Furthermore, DKK and soluble Frizzled-related protein (sFRP) binds to LRP5/6 and Fzd (Figure 3) [61], sequentially, and block the formation of the LRP-Wnt-Fz complex in reaction to Wnts. Synaptic injury precedes neuronal death in the neurodegenerative phase of Alzheimer’s disease. The Wnt/β-catenin signaling pathway is one of the most important regulators of cell death and life. Conversely, loss of Wnt/-βcatenin signaling makes neurons increasingly vulnerable to Aβ-induced apoptosis, whereas amplification of Wnt/β-catenin signaling prevents neurodegeneration and behavioral impairments caused by Aβ. In contrast, the amplification of Wnt/β-catenin signaling prevents neurodegeneration and behavioral impairments caused by Aβ [44,46].

While the existence of neurogenesis inside the human adult brain is debated, new data reveal that human hippocampus neurogenesis continues in older adults and falls drastically in AD patients. Wnt/ β -catenin signaling is a major regulator of adult hippocampus neurogenesis, according to a growing body of research. By boosting Wnt/β-catenin signaling and particular downstream target genes involved in cell cycle control and neural development [46,65], Wnt-7a plays a very important role in many stages of neurogenesis [65]. Moreover, in aged mice, astrocyte-secreted Wnt proteins are reduced, resulting in Wnt/ β-catenin signaling regulation, down-regulation of survivin levels in neural progenitor cells (NPCs), and reduced adult regeneration. Neural activity caused by an anti-aggregant tau mutant is coupled with Wnt/ β -catenin signaling activation, which is noteworthy [44]. The activation of the Wnt/β-catenin signaling pathway is required for transcriptional activation of the mitotic regulator survivin, the basic helix-loop-helix transcription factor Neuro-D1 [42], as well as the prosper-related homeobox transcription factor Prox-1. All of the aforementioned are required for the generation of granule cells in the hippocampus. We outline our modern knowledge of the involvement of Wnt/β-catenin signaling in many physiological and pathological processes in the AD brain in the remainder of this section [66].

## 5. The Blood–Brain Barrier (BBB) and Its Dysfunction in the Pathogenesis of AD

For the regulation and differentiation of the central nervous system, to control its energy supply and permeability, the BBB acts as a tight control. Formed by the cerebral microvascular endothelium, whose tight junctions (TJs) control the paracellular transport of hydrophilic and charged substances [67], the BBB ensures the maintenance of homeostasis in the central nervous system [68]. Migration of immune cells across the BBB or blood–cerebrospinal fluid barrier (BCSDB) into the cerebrospinal fluid (CSF)-drained spaces of the CNS, followed by progression across the glia limitans, or glial limiting membrane into the CNS parenchyma, are two differing regulated steps by which immune cells can overcome the specialized structure of the BBB [69]. In research, the focus is primarily on elucidating the different molecular mechanisms required for the migration of immune cells across the various CNS barriers in multiple sclerosis. In this context, other neuroinflammatory diseases and comorbidities still need to be thoroughly investigated for their mechanism.

Alzheimer’s disease (AD) is a neurodegenerative disorder that has cerebral effects such as neurovascular dysfunction [70], cognitive decline, accumulation of amyloid-β-peptide-Aβ, and also tau-related lesions in neurons, also called neurofibrillary tangles [71]. The risk factors for cerebrovascular disorders and sporadic AD have remarkable overlap, as several epidemiological studies have shown. The risk of AD, as well as vascular dementia, is increased by, for example, diabetes in middle age, hypertension, and obesity [72,73]; in addition, mixed vascular pathology and small vessel disease is present in most AD cases [74]. The risk of AD is also increased by reduced cerebral perfusion, silent infarcts, and the presence of one or more infarcts [74,75].

A cascade triggered by Aβ results in neuronal damage and loss of nerve cells, accompanied by cognitive decline, according to the amyloid hypothesis [76]. Following the vascular two-stroke hypothesis of Alzheimer’s disease, vascular damage occurs first (stroke 1), followed by a second insult (stroke 2) due to Aβ accumulation in the brain [77]. As discussed below, all vascular factors could amount to a common disease process with microvascular dysfunction and/or degeneration of the brain, as well as Aβ and tau pathology, although the molecular and cellular events are not completely clear for each step in the disease process nor for each risk factor. Based on the vascular hypothesis, reduced cerebral blood flow (CBF) and hypoxia [78], on the one hand, but also blood–brain barrier (BBB) dysfunction with cerebral accumulation of vasculotoxic and neurotoxic macromolecules [79], on the other, may lead to neuronal dysfunction and neurodegenerative changes occurring independently and/or before Aβ deposition [73,78]. Defective Aβ clearance from the brain increased influx of peripheral Aβ across the BBB and/or increased expression of β-amyloid precursor protein (APP) [80]; their origin may lie in cerebrovascular dysfunction and injury, as several studies have shown [80]. This leads to Aβ accumulation in the brain and around the cerebral blood vessels [81]. As in prion diseases, increased Aβ concentrations in the brain can advance neurovascular and neuronal dysfunction, as well as promote self-propagation, leading to cerebral β-amyloidosis [82].

Other hypotheses suggest that environmental risk factors such as smoking and infection are involved. As mentioned earlier, this neurovascular hypothesis states that the blood–brain barrier, which is crucial for Aβ homeostasis in the brain, regulates Aβ transport via the LRP receptor [83] and RAGE [84]. For AD, these findings may be clues to new therapeutic strategies.

## 6. Wnt-β Signaling and Functionality of Blood–Brain Barrier in AD 

The BBB shields the brain from harmful blood-derived detritus, cells, and microbial pathogens [85]. As a result, BBB breakdown permits hazardous compounds to enter the brain, triggering inflammatory and immune responses, and potentially initiating various neurodegenerative processes [86,87]. In Alzheimer’s disease, BBB degradation is an early indicator of human cognitive decline. It is discovered prior to the onset of schizophrenia, neurodegeneration, or brain atrophy [88,89]. Increased BBB vulnerability, micro-bleeding, decreased glucose transport, impaired Pgp-1 activity (alpha–beta clearance), perivascular build-up of neurotoxic blood-derived products, and cellular infiltration and degradation of pericytes and endothelial cells are all pathogenic features of AD [90]. As a result, exploring innovative methods for BBB repair is a viable option for treating Alzheimer’s disease. The Wnt/β-catenin pathway has been identified as a crucial network essential not only for BBB development, but also for BBB preservation and activity in recent years [91,92]. Wnt ligands Wnt-7a and Wnt-7b, which are produced mainly by neurons and astrocytes in the brain, activate Wnt/β-catenin signaling in BBB endothelial cells (ECs) by binding to Wnt receptor Fzd-4 and Wnt co-receptor LRP5/6; Wnt/β-catenin signaling is the main factor of BBB construction and maintenance [92,93]. Additionally, Reck, a GPI-anchored membrane protein, and Gpr-124, an orphan GP-CR, are required cofactors on the surface of the cell for Wnt7a/Wnt7b signaling in human CNS angiogenesis and BBB preservation and performance [94]. Tight junctions that are primarily made up of claudins link brain ECs together. Furthermore, glucose transporter-1 (GLUT-1) [95], which is abundantly expressed on the endothelium of BBB endothelial cells, is necessary for the transport of glucose from the blood into the brain; and pglycoprotein (Pgp-1) is an active efflux transporter found to be overexpressed on the luminal surface of BBB endothelial cells [96,97]. The three primary claudins expressed in brain ECs, claudin-1-3-5, are transcriptional targets of Wnt/beta-catenin signaling in BBB ECs mechanically [98]. Importantly, Wnt/beta-catenin signaling in BBB ECs promotes the development of the BBB-specific glucose transporter GLUT-1 and the efflux transporter Pgp-1 [99].

**Figure 4:**
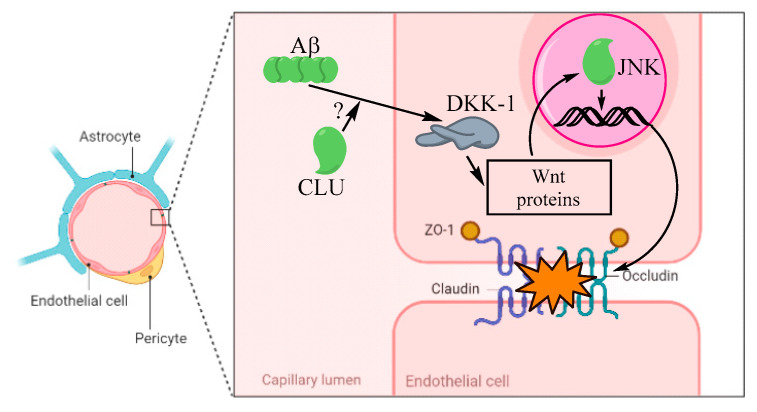
The intracellular *CLU*-mediated Aβ toxicity at the BBB via an unknown mechanism.

Moreover, Killick et al. discovered that knockdown of *CLU* in primary neurons using penetrating peptide (Pen1)-coupled siRNA duplex with *CLU* reduces Aβ toxicity [100]. Similarly, the *CLU* gene has recently been identified as a risk factor for AD [101]. Figure 4 illustrates how the Aβ toxicity at the BBB could be mediated by *CLU* directly or indirectly via an unknown mechanism. This can be triggered by the activation of the DKK-1/Wnt pathway followed by the subsequent activation of JNK in the nucleus. The latter molecule promotes the expression of pro-apoptotic genes. Finally, the accumulation of cytotoxic substances in a cell promotes damage to tight junctional proteins (ZO-1, claudin, ccluding), leading to increased BBB permeability (Figure 4).

## 7. Cellular Risk Aspects of *CLU* Protein in AD (Lipid Metabolism, Homeostasis, Neuronal Apoptosis)

*APOJ*, the brain’s second most important apolipoprotein, is encoded by *CLU*. Furthermore, it shares the majority of *APOE*’s features, not only in terms of alpha–beta, but also in aspects of lipid transport [60,102]. Likewise, it is linked to the transport of phospholipids and cholesterol.

In turn, high clusterin levels have been linked to atherosclerosis. *CLU* was also discovered to be involved in the efflux of cholesterol from lipid-loaded murine macrophages [103]. Carotid intima-media thickness and lipid levels have just been linked to apolipoprotein J polymorphisms. This suggests that *CLU* genetic differences may affect AD vulnerability indirectly by increasing the risk of cerebrovascular illness, which can lead to neurodegeneration [102,104]. Human brain matter contains an above-average level of myelin lipids covering axons and cellular membranes [103]. Since lipids are insoluble, they cannot be moved across cells that are not adjacent until they are solubilized and carried in soluble lipoprotein particles. Apolipoprotein J is a key cholesterol transport lipoprotein in the brain [103,105]. The effects of cholesterol on Alzheimer’s disease pathogenesis indicate the actions of the lipoproteins-based brain cholesterol transport in lipoprotein particles. They also demonstrate that lipid metabolism alters the activities contained within Aβ-related processes [54,106]. More research is needed to determine if the appearance of the clusterin polymorphism immediately moderates the metabolic changes occurring throughout the process of a disease. Moreover, it indirectly impacts brain lipid metabolism by way of amyloid actions or possible actions within the cerebrovascular system [60].

One of the events of AD pathogenesis is known to be altered copper homeostasis. The association between copper ATPases and *CLU* was observed to increase as a result of ATP7B mutations and oxidative stress. It would imply that peroxidation caused by low copper levels may be mediating *CLU*-linked ATP-7A and B degradation [107]. The two SNPs in moderate linkage disequilibrium within ATP7B (rs_732774 and rs_1061472) at the place where haplotype had been discovered located in the ATP-7B areas encoding for functionally relevant transmembrane and transduction regions have remained connected to a bigger risk of Alzheimer’s disease [102,104,107].

Glial activation is one of the early mechanisms at AD onset and is assumed to be a pathophysiological response to increased Aβ deposition [108]. This has been shown to impact the clinical evolution of cognitive decline in AD patients [109]. Reactive astrocytes, also called reactive astrogliosis, can be demonstrated by the assessment of specific fluid biomarkers such as glial fibrillary acidic protein (GFAP), S100B, and chitinase-3-like protein 1 (YKL-40) [110]. This was shown to affect several physiological pathways and AD phenotypes, as it triggers morphological, functional, and molecular changes, e.g., tau pathology or glucose consumption, and pro-inflammatory pathways.

Nuclear *CLU* (*nCLU*) is pro-apoptotic, although secreted *CLU (sCLU)* is pro-survival. Both of these *CLU* forms have been linked to a variety of biological functions, including cellular cycle progression, apoptotic cell mortality, along with DNA reparation [111,112]. Numerous studies have shown that induced overexpression of full-length apolipoprotein J mRNA can cause non-physiological *nCLU* production, which can act as a signal corresponding to pro-cell death, resulting in cellular growth and mortality [112,113]. Interestingly, *nCLU* deletion and *sCLU* overexpression were revealed to be associated with tumor cell longevity. The latest discoveries revealed that cells must inhibit *sCLU* to promote cell death, which backed up this theory [111,114]. *CLU* has also been linked to DNA repair signaling, especially in the non-homologous end-joining pathway. Indeed, the *nCLU* protein can form a trimeric protein complex with Ku-80 by binding to Ku70 [111,112]. Overexpression of *nCLU* reduces the ability of Ku-80/Ku-70 to bind to DNA in whole-cell extracts. *CLU* also plays an important role in controlling cell cycle progression. Forced full-length *CLU* mRNA overexpression (due to *nCLU* expression) resulted in an increased accumulation of cells in the G0/G1 phases of the cell cycle, as well as decreased DNA production and cell cycle progression in immortalized human prostate cancer cells [112]. Increased *sCLU* levels caused G1 cell cycle arrest in a range of cell types. Moreover, it also includes DNA repair, which is crucial in cases corresponding to Alzheimer’s disease. As a result of the imbalance in these regulative processes, they were linked to the development of AD [112,115].

## 8. Conclusions

Several pathways have been discovered possibly explaining *CLU*’s pathogenic function in Alzheimer’s disease. *CLU* has been associated with a multitude of other mechanisms. This is in addition to brain cholesterol and lipid metabolism levels, neuro-inflammation, impaired Wnt signaling, the BBB System, and copper-based homeostasis, and the repression of neuronal apoptotic cell death of neuroprotective characteristics. The aforementioned could be linked or explained due to its possible affiliations with Aβ clearance and aggregation. Routes linked to AD pathogenesis must be discovered from these mechanisms to create effective AD therapeutics. Since it has a diverse spectrum of activities and isoform-specific actions, *CLU-APOJ* should not be viewed as a standard target. Nonetheless, this could be advantageous for multivariate analysis of AD. 

## Data Availability

Not applicable.
